# Future physician-scientists: could we catch them young? Factors influencing intrinsic and extrinsic motivation for research among first-year medical students

**DOI:** 10.1007/s40037-018-0440-y

**Published:** 2018-07-13

**Authors:** Belinda W. C. Ommering, Floris M. van Blankenstein, Cathelijn J. F. Waaijer, Friedo W. Dekker

**Affiliations:** 1 Center for Innovation in Medical Education, Leiden University Medical Center, Leiden, The Netherlands; 2 Department of Higher Education, Leiden University Graduate School of Teaching, Leiden, The Netherlands; 3 Department of Clinical Epidemiology, Leiden University Medical Center, Leiden, The Netherlands

**Keywords:** Undergraduate research, Motivation, Physician-scientists, Medical education

## Abstract

**Introduction:**

The medical field is currently facing a physician-scientist shortage. One possible solution is to direct medical students towards a research oriented career. To do so, knowledge is needed on how to motivate medical students to do research. Therefore, this study examines motivation for research and identifies factors influencing intrinsic and extrinsic motivation for research among first-year medical students.

**Methods:**

First-year medical students were surveyed at the beginning of their bachelor’s program in 2016. On a 7-point Likert scale, students reported their motivation for research, self-efficacy, perceptions of research, curiosity, and need for challenge. Regression analyses were used to examine the influence of these factors on students’ motivation for research.

**Results:**

Out of 316 approached students, 315 participated (99.7%). On average, students scored 5.49 on intrinsic, and 5.66 on extrinsic motivation for research. All factors measured influenced intrinsic and extrinsic motivation for research significantly and positively, also after adjusting for gender and age. Cumulative regression showed that these factors explained 39.6% of the variance in intrinsic, and 14% in extrinsic motivation for research.

**Discussion:**

All factors play an important role in intrinsic and, to a lesser extent, extrinsic motivation for research. First-year medical students’ motivation for research could be enhanced by stimulating positive self-efficacy beliefs, positive perceptions of research, and curiosity. Also, it is important to fulfil students’ needs for challenge by stimulating them to actively conduct research. Thus, to catch students young and cultivate physician-scientists, students should be stimulated to engage in research from the beginning of medical training.

## What this paper adds

This paper builds on existing literature that advocates engaging medical students in research to counteract the decline of physician-scientists. Studies focusing on motivating medical students for research are scarce, and mainly concentrate on clinical phases. Moreover, those studies often lack a sound theoretical framework. We used Self-Determination Theory to investigate motivation for research among first-year medical students. Our findings show that first-year medical students are already motivated for research and that their motivation is influenced by self-efficacy, perceptions of research, curiosity, and need for challenge. This offers possibilities to ‘catch students young’ and stimulate early engagement in research to cultivate physician-scientists.

## Introduction

According to the Canadian Medical Education Directives for Specialists (CanMEDS), all doctors should be able to critically appraise and use research in clinical practice to form decisions and make a grounded diagnosis [1]. Furthermore, it is necessary for doctors to keep up with current developments within their field of expertise [2]. To use research and apply evidence-based practice doctors should be able to understand research [3–6].

Not only should all physicians use research, there is also a need for doctors to conduct research. Research can contribute to the creation of new knowledge, which is necessary to keep doctors up-to-date and to make progress in the dynamic world of medical healthcare [4, 5, 7]. Physician-scientists can bridge the gap between science and practice, by translating research outcomes into clinical settings [8–11]. Moreover, physician-scientists encounter actual relevant clinical questions and problems, which can serve as inspiration for scientific research [12].

Currently, there is a shortage in the number of physician-scientists, as too few doctors pursue a scientific career [1, 8, 11, 13–15]. In a recent review, Chang and Ramnanan stated that in Europe, the United States, and Canada, interest in research among physicians is still decreasing [1]. Milewicz and her colleagues published a report in 2015 showing that too few young physicians pursue a scientific career, stressing the urgent need to direct more physicians towards research [16]. To summarize, the medical field is developing rapidly, and consensus exists on the urgent need for more physician-scientists. However, how future doctors can be stimulated to pursue a scientific career is still debated [4, 8, 9, 17–21].

Recent literature suggests that early engagement of medical students in research might be an effective solution [1–5, 11, 21, 22]. This may not only help to trigger enthusiasm and stimulate future engagement in research [1, 23, 24], but may also help to recognize talent and to help this talent develop into physician-scientists [25].

To stimulate medical students for and keep them interested in research, it is important to know what motivates them for doing research in early phases of medical training and which factors contribute to their motivation for research. Most medical students in the Netherlands start their medical education after graduating from high school, at the age of 18–20 years. It is unknown if these young students recognize the importance of doing research, and in what way they are motivated to do research.

With regard to motivation in general, studies have shown that medical students are highly motivated, because of their large investment in entering medical school [26]. Less is known about their motivation specifically for research and studies relying on a sound theoretical framework are scarce. One study using Self-Determination Theory (SDT) indicated that students view research as valuable for their future medical career [3]. However, these results were mostly applicable to students in clinical years, and less to students in pre-clinical phases.

SDT distinguishes two main types of motivation: intrinsic and extrinsic motivation. Furthermore, SDT identifies three basic psychological needs influencing motivation: the need for competence, autonomy, and relatedness [26, 27]. In this study, we focus on the outcome measures of the SDT, namely intrinsic and extrinsic motivation. In the context of medical education, students can be *extrinsically* motivated to do research because it is beneficial for future training and career opportunities, for example to secure a competitive residency spot [1, 11, 28–30]. Additionally, there is evidence that students can be *intrinsically* motivated for research, and participate out of interest and enjoyment [11, 28, 29]. Students could also have both intrinsic and extrinsic motives for doing research [31].

We investigated four factors that may influence intrinsic and extrinsic motivation to conduct research at the start of medical training. The first factor, *self-efficacy*, is a person’s belief in his or her own ability to accomplish a certain outcome [32]. Studies indicated that people are more inclined to follow a certain path if they are confident in their own capability in that domain, and that self-efficacy for research contributes to motivation for a research oriented career [32–34]. In this study, we explored students’ beliefs in their general capabilities (i. e. general self-efficacy), academic capabilities (i. e. academic self-efficacy), and research-related capabilities (i. e. research self-efficacy). Previous studies suggested that higher levels of general and academic self-efficacy increase students’ engagement in challenging tasks [35]. Doing research is considered to be a complex, and therefore challenging task, especially for first-year students. Therefore all three types of self-efficacy might play a role in influencing motivation for research.

The second factor we investigated was *perceptions of research*: students’ beliefs about the value of research and learning. Perceptions of research as a predictor of motivation for research has not yet been investigated. However, a relation between these factors seems plausible. For instance, positive perceptions of research might co-occur with higher motivation for research. If so, it could be valuable to promote positive perceptions of research during medical training.

The third factor we investigated was *curiosity*: the desire to gain new knowledge. Berlyne introduced the concept of ‘epistemic curiosity’ and described this as ‘the drive to know’ (1954, P. 187) [36]. Epistemic curiosity can be divided into two types: interest curiosity concerns the satisfaction in discovering new ideas, and deprivation curiosity concerns the effort spent on finding solutions to a problem [37]. In medical education, students are stimulated to ask questions in order to enhance learning (i. e. interest curiosity) [6]. Additionally, students have to solve problems when they encounter difficulties and unknown areas (i. e. deprivation curiosity). Previous research showed that curiosity underlies motivation for and participation in research [22, 24, 25]. We investigated which type of curiosity was more important in affecting motivation for research. This could provide insights into which type of curiosity should be encouraged explicitly during medical training.

The fourth factor we investigated was *need for challenge*, specifically the need for extracurricular challenges. Some students need extra challenges, which, if not satisfied, could lead to drop out or lower academic performance [38]. If need for challenge influences motivation for research, educators can stimulate students by having them participate in research, for instance by offering challenging research projects.

To investigate if students are motivated to conduct research and which factors influence motivation, we posed the following research questions: 1) to what extent are first-year medical students intrinsically and/or extrinsically motivated for research; and 2) what influence do self-efficacy, perceptions of research, curiosity, and need for challenge have on intrinsic and extrinsic motivation for research among first-year medical students? If we know the nature of students’ motivation for research and which factors influence motivation for research, evidence-based strategies can be implemented to enhance medical students’ interest in research, and the first step to educate the next generation of physician-scientists can be made. Moreover, it could also have implications for the recruitment of students for medical training, as it could provide insights into how to attract possible future physician-scientists.

## Methods

### Participants

This study surveyed first-year medical students at Leiden University Medical Center. All students starting their medical education (bachelor’s program) in 2016 were asked to participate in this survey.

### Materials

A 7-point Likert type questionnaire consisting of 36 items was composed, ranging from 1 (totally disagree) to 7 (totally agree). We adjusted existing and validated scales in order to focus on research activities and the medical education setting. The scales, reliability, and sample items of the factors as measured by the questionnaire are shown in Tab. 1. Motivation for research was divided into questions regarding intrinsic and extrinsic motivation. Items for both types of motivation were based on Self-Determination questionnaires. Intrinsic motivation was based on the Interest/Enjoyment Scale [27, 39], and extrinsic motivation on the Value/Usefulness Scale [27, 39]. Self-efficacy was divided into questions regarding general, academic, and research self-efficacy. For measuring general self-efficacy, the Dutch General Self-Efficacy Scale was used [40]. For academic self-efficacy, the Academic Efficacy Scale from the Patterns of Adaptive Learning Scales (PALS) was used [41]. Items on the research self-efficacy scale were self-developed and designed based on the previous two efficacy scales, but more specifically addressing self-efficacy regarding research. Perceptions of research were examined by using the subscale from the Student Perception of Research Integration Questionnaire (SPRIQ) focusing on students’ beliefs about the value of research and learning [42]. Curiosity was measured with the Epistemic Curiosity Scale, divided in items on interest and deprivation curiosity [37]. Need for challenge was studied with self-developed items.

**Table 1:** Scales, reliability, and sample items of the questionnaire used in this study

Scale^a^	*N* items	Cronbach’s *α*	Sample item
Intrinsic motivation	5	0.79	Doing research is fun
Extrinsic motivation	4	0.77	I think doing research improves my chances for my preferred residency spot
General self-efficacy	3	0.78	I trust my ability to solve problems
Academic self-efficacy	3	0.84	If I try I can deal with the most difficult parts of the course
Research self-efficacy	3	0.88	I feel I am competent enough to do research
Perceptions of research	5	0.83	It is important for medical professionals to have scientific skills
Interest curiosity	5	0.80	I enjoy investigating new ideas
Deprivation curiosity	5	0.84	If I am busy with a problem, I won’t rest until I have the answer
Need for challenge	3	0.81	I desire an extra challenge on top of the curriculum

^a^All items were answered on a 7-point Likert scale

### Procedure

The questionnaire was translated from English to Dutch, using the forward and backward translation procedure, and pretested on ten second-year medical students. Based on this pilot study, two items were clarified with minor adjustments in the use of words, and all first-year medical students were surveyed. Students were approached by the first author at the beginning of a workgroup session and asked to fill out the questionnaire. They were told that the study was to investigate scientific education in the medical bachelor program, participation was voluntary, and all data would be processed anonymously. Students who agreed to participate received the questionnaire. This study was approved by the educational institutional review board of Leiden University Medical Center: IRB reference number OEC/OG/20161108/2.

### Analysis

We used descriptive statistics to describe demographic variables and previous educational experiences of the students. To estimate the reliability of the scales in the questionnaire, we calculated Cronbach’s alpha (Tab. 1). We calculated mean scores for every scale (range 1 to 7) and used independent t‑tests, with Bonferroni correction, to study possible differences between male and female students. To test which factors influence motivation for research, we used univariate linear regressions, adjusted for gender and age. We applied a 95% confidence interval. Based on existing literature we also constructed a cumulative model of explained variance (*R*^*2*^), starting with the most frequently investigated factors. We analyzed all data using IBM SPSS Statistics version 23 for Windows.

## Results

Of all 316 students who were approached, 315 students agreed to participate in this study (99.7%). The study included 90 male (28.6%) and 225 female participants (71.4%). Most of the students started medical education immediately after graduating from high school (86.3%), resulting in a sample with a mean age of 18.57 years (*SD* = 1.37). Of these students, 30.2% indicated they had some kind of previous experience with participating in research.

Medical students were intrinsically (*M* = 5.49, *SD* = 0.79) as well as extrinsically (*M* = 5.66, *SD* = 0.80) motivated for research, as can be seen in Tab. 2 with the descriptives of the sample. Of all students, 30.1% scored a 6 or higher on intrinsic motivation and 42.5% scored a 6 or higher on extrinsic motivation for research. Analysis showed that female students scored slightly higher on both intrinsic and extrinsic motivation for research, but this was not statistically significant (*p* = 0.14 and *p* = 0.07 respectively). The independent t‑tests showed that male students scored significantly higher than female students on general self-efficacy (*p* < 0.001, male students scoring 0.39 higher), and academic self-efficacy (*p* < 0.001, male students scoring 0.45 higher). These remained significant after Bonferroni correction (9 tests performed; α < 0.05/9 = 0.0056). No significant differences between male and female students were found on research self-efficacy (*p* = 0.33), perceptions of research (*p* = 0.50), interest curiosity (*p* = 0.61), deprivation curiosity (*p* = 0.35), and need for challenge (*p* = 0.55).

**Table 2:** Description of scores on factors measured in the questionnaire^a^

	*N*	Mean	SD	Min	Max	≥6 (%)
Intrinsic motivation	314	5.49	0.79	2.8	7.0	30.1
Extrinsic motivation	315	5.66	0.80	3.0	7.0	42.5
General self-efficacy	315	5.48	0.76	2.7	7.0	35.2
Academic self-efficacy	315	5.43	0.93	2.3	7.0	36.4
Research self-efficacy	314	4.85	0.97	2.0	7.0	18.1
Perceptions of research	315	5.53	0.81	2.4	7.0	34.9
Interest curiosity	315	5.46	0.77	3.2	7.0	28.1
Deprivation curiosity	315	4.80	1.02	1.6	7.0	16.2
Need for challenge	314	4.10	1.18	1.0	7.0	0.6

^a^Based on a 7-point Likert scale

Univariate linear regression analysis indicated that all factors influenced intrinsic and extrinsic motivation for research significantly, as can be seen in Tab. 3. The associations remained significant after adjusting for gender and age. All regression coefficients were higher for intrinsic motivation as compared with extrinsic motivation for research.

**Table 3:** Effects on intrinsic and extrinsic motivation: crude and adjusted for gender and age^a^

	Intrinsic motivation		Extrinsic motivation	
	Crude β(95% CI)	Adjusted β(95% CI)	Crude β(95% CI)	Adjusted β(95% CI)
General self-efficacy	0.257(0.154–0.360)	0.298(0.192–0.404)	0.169(0.053–0.285)	0.213(0.095–0.331)
Academic self-efficacy	0.217(0.132–0.302)	0.320(0.168–0.340)	0.123(0.028–0.218)	0.156(0.059–0.253)
Research self-efficacy	0.370(0.297–0.444)	0.384(0.310–0.457)	0.199(0.109–0.288)	0.209(0.120–0.298)
Perceptions of research	0.430(0.339–0.520)	0.438(0.350–0.527)	0.330(0.226–0.433)	0.338(0.235–0.441)
Interest curiosity	0.447(0.354–0.540)	0.444(0.350–0.539)	0.252(0.140–0.365)	0.250(0.138–0.362)
Deprivation curiosity	0.256(0.182, 0.331)	0.245(0.169–0.320)	0.187(0.102–0.271)	0.182(0.097–0.267)
Need for challenge	0.310(0.250–0.370)	0.316(0.257–0.376)	0.189(0.117–0.262)	0.191(0.119–0.264)

^a^All *p*-values were below 0.05, all but three *p*-values were below 0.01

The cumulative linear regression model indicated that self-efficacy explained 25.1% of the variance in intrinsic motivation for research. In this cumulative model, curiosity added 7.8% in explaining the variance, and if need for challenge and perceptions of research were included a total of 39.6% of the variance in intrinsic motivation for research can be explained, as illustrated in Tab. 4. With regard to extrinsic motivation for research, the total variance explained is 14%, of which self-efficacy contributed 6% and the other factors all together explained 8%.

**Table 4:** Cumulative model of explained variance of intrinsic and extrinsic motivation for research

		Intrinsic motivation				Extrinsic motivation			
Model	Variable	β	SE	*p*	cum. R^²^	β	SE	*p*	cum. R^²^
1	Research self-efficacy	0.495	0.037	0.000	0.245	0.239	0.045	0.000	0.057
2	Research self-efficacy	0.459	0.041	0.000	0.250	0.219	0.050	0.000	0.059
	Academic self-efficacy	0.083	0.043	0.128		0.045	0.053	0.461	
3	Research self-efficacy	0.464	0.044	0.000	0.251	0.208	0.053	0.001	0.060
	Academic self-efficacy	0.094	0.052	0.153		0.022	0.063	0.762	
	General self-efficacy	−0.020	0.066	0.770		0.043	0.080	0.572	
4	Research self-efficacy	0.375	0.043	0.000	0.329	0.162	0.055	0.015	0.081
	Academic self-efficacy	0.048	0.050	0.440		−0.001	0.063	0.984	
	General self-efficacy	−0.062	0.062	0.336		0.021	0.080	0.782	
	Interest curiosity	0.316	0.050	0.000		0.164	0.064	0.008	
5	Research self-efficacy	0.372	0.043	0.000	0.329	0.144	0.055	0.033	0.088
	Academic self-efficacy	0.050	0.050	0.426		0.014	0.064	0.854	
	General self-efficacy	−0.064	0.063	0.328		0.011	0.080	0.882	
	Interest curiosity	0.307	0.061	0.000		0.098	0.077	0.187	
	Deprivation curiosity	0.015	0.044	0.802		0.114	0.056	0.114	
6	Research self-efficacy	0.304	0.043	0.000	0.374	0.099	0.057	0.149	0.107
	Academic self-efficacy	−0.032	0.050	0.615		−0.040	0.066	0.600	
	General self-efficacy	−0.023	0.061	0.717		0.038	0.080	0.620	
	Interest curiosity	0.231	0.061	0.000		0.049	0.079	0.523	
	Deprivation curiosity	−0.027	0.043	0.657		0.086	0.056	0.231	
	Need for challenge	0.277	0.037	0.000		0.179	0.048	0.011	
7	Research self-efficacy	0.258	0.044	0.000	0.396	0.043	0.057	0.537	0.140
	Academic self-efficacy	−0.037	0.050	0.553		−0.046	0.064	0.537	
	General self-efficacy	−0.024	0.060	0.705		0.037	0.078	0.621	
	Interest curiosity	0.181	0.062	0.005		−0.012	0.080	0.873	
	Deprivation curiosity	−0.030	0.043	0.618		0.083	0.055	0.242	
	Need for challenge	0.243	0.037	0.000		0.138	0.048	0.050	
	Perceptions of research	0.184	0.050	0.001		0.225	0.065	0.001	

## Discussion

This study showed that first-year medical students are already motivated to do research, as they score relatively highly on both intrinsic and extrinsic motivation. Results also show that self-efficacy, perceptions of research, curiosity, and need for challenge are all positively associated with intrinsic and extrinsic motivation for research, also after adjusting for gender and age. The cumulative regression model indicated that around 40% of the variance in intrinsic motivation for research can be explained by the factors included in this study, especially research self-efficacy, interest curiosity, need for challenge, and perceptions of research were important. With regard to extrinsic motivation for research, only 14% of the variance was explained by the factors measured.

On a scale of 1–7 to indicate motivation for research, students scored on average above a 5, which implies that the group was both intrinsically and extrinsically motivated. This is an interesting finding, as one could assume that new medical students would be particularly interested in becoming a clinician. For instance, Rosenkranz and colleagues [3] showed that students in medical training acknowledged the relation between research and keeping up to date, but it was not until they experienced uncertainties in clinical practice that they understood the real relevance of research. A claim that the authors make is that medical students want to be clinicians, and that feelings of the importance of a good doctor conducting research appear in the clinical years of medical training [3]. Our results indicate that our students are already motivated and can see the importance of research at the beginning of their medical training.

Our findings are in line with previous studies in showing that self-efficacy contributes to motivation for research. It has been suggested that doctors with high research self-efficacy are more inclined to pursue a scientific career [34]. Our study suggests that this might also be the case for medical students, with higher research self-efficacy enhancing motivation of students for research this early in medical education. This is in line with Bandura’s Social Cognitive Theory, which says that self-efficacy has a critical influence on motivation in general [32]. Our study provides support for the applicability of the Social Cognitive Theory in more specific settings, such as motivation for doing research.

Whereas all types of self-efficacy were positively related to both intrinsic and extrinsic motivation for research if tested separately, cumulative testing revealed that general and academic self-efficacy did not contribute to motivation on top of research self-efficacy. It has been argued that first-year medical students see research as a very specific task where very distinct skills are needed [43], which could mean that the items related to research self-efficacy are more concretely linked to research and thus motivation for research in this sample. Our results indicate that it is valuable to promote positive research self-efficacy beliefs in medical students. Ambiguity and uncertainty regarding an unknown activity may cause lower self-efficacy beliefs [32]. By providing students with more research related experiences early in the curriculum adapted to their level, students can become familiar with doing research. This is also in line with the Social Cognitive Theory, which states that mastery of an activity leads to higher self-efficacy beliefs [32]. With the right support, positive research self-efficacy beliefs can be stimulated, which can contribute to students’ motivation for research.

In contrast to earlier studies [42–44], this study examined perceptions of research as the independent variable. Our results showed that perceptions of research strongly influenced intrinsic and extrinsic motivation. One could argue that students may not be motivated to do research if it does not seem directly valuable for their development (i. e. intrinsic motivation) or future career (i. e. extrinsic motivation). This result could offer great opportunities because perceptions can be influenced [42, 43]. By stimulating positive perceptions of research, motivation for research can be enhanced, thus it seems important to structure medical education in a way that positive perceptions are promoted. This could be done by exposing students to conducting research, and emphasizing its relevance for future clinical practice.

With regard to curiosity, the results in this study are in line with earlier findings showing that curiosity influences motivation for research [22, 24, 25]. It could be that curiosity reflects some kind of eagerness or ambition that underlies motivation, regardless of whether the nature of motivation is intrinsic or extrinsic. Our results indicate that interest curiosity, as well as deprivation curiosity, positively influence motivation for research. Both types of curiosity seemed to matter, but interest curiosity played a greater role in explaining differences in motivation for research. It is desirable to continue to stimulate students’ curiosity.

Lastly, our findings showed that some medical students are in need of extra challenges and that this relates to their motivation for research. This indicates the importance of identifying students in need of extra challenges, in order to get them acquainted with the possibility to conduct research, thereby adding research to their options to meet with their need for challenge. In this way, identifying students in need of extra challenges may help to counter the physician-scientist shortage.

Milewicz and her colleagues showed that a MD-PhD program is a successful approach to train physician-scientists, and argued that this may be extended to postgraduate training as well [16]. Our results, however, suggest that these efforts could be pointed at much younger medical students too, by integrating research much earlier into medical training. Since first-year students are already motivated for research this early in medical training, it is our responsibility as educators to make sure that this motivation does not evaporate.

### Limitations and strengths

A first limitation of this study is its cross-sectional design. It could be valuable to measure the factors at different time points to establish a deeper knowledge regarding how they relate to each other and to both types of motivation for research over time. Secondly, in our study we distinguish two types of motivation for research: intrinsic and extrinsic. SDT distinguishes these two types of motivation in the same way, but a refined version of this theoretical framework shows extrinsic motivation as a spectrum with four types of extrinsic motivation, varying in the quantity of external influences [26]. The items we used to compose the scale to measure extrinsic motivation are mostly related to the external and introjected regulation category. Future research would benefit from refining the concept of extrinsic motivation for research and investigating which factors influence what types of extrinsic motivation. Thirdly, motivation was self-reported and it is unclear whether students will act on their motivation by actually participating in research. Strengths of this study are the large sample size including almost all first-year medical students in our university medical centre and the high reliability of the scales (scales of 3–5 items, α > 0.77). This extent of participating students and the large sample size ensures that this study forms a sound base for investigating what influences motivation for research among first-year medical students.

### Conclusion

Students’ motivation for research could be enhanced by arranging the medical curriculum in a way that continuously stimulates positive self-efficacy beliefs, positive perceptions of research, and curiosity. Besides, we should be aware of and foster students’ need for extra challenge by stimulating them to participate in research. Educators should emphasize the importance of conducting research for future clinical practice in such ways that students feel that it is valuable to fulfil their need for challenge by conducting research. Thus, this offers possibilities to catch them young and thereby contributes to the future physician-scientists workforce. The results of this study have shown that students are motivated for research early in medical training and therefore it is our duty to foster these students’ motivation.
